# IMiDs for myeloma induced renal impairment

**DOI:** 10.18632/oncotarget.26270

**Published:** 2018-10-26

**Authors:** Maria Gavriatopoulou, Evangelos Terpos, Meletios Athanasios Dimopoulos

**Affiliations:** Department of Therapeutics, Alexandra General Hospital, National and Kapodistrian University of Athens, School of Medicine, Athens, Greece

**Keywords:** multiple myeloma, renal impairment, thalidomide, lenalidomide, pomalidomide

Renal impairment (RI) is one of the most common complications in patients with multiple myeloma (MM) and is present in approximately 20% at diagnosis and 40%-50% during the disease course. The presence of moderate and severe RI is associated with poorer outcomes and increases mortality and morbidity rates, as well as the risk of early death. Therefore, rapid intervention and antimyeloma treatment is essential. The management of patients with RI still remains a challenge for the hematologists. Several drugs cannot be used due to potential nephrotoxicity, while their dosage should be closely adjusted and renal function closely monitored. Treatment initiation aims to disease improvement including renal function response. Although bortezomib-based regimens remain the standard of care for these patients immunomodulatory agents (IMiDs) such as thalidomide, lenalidomide and pomalidomide may be used either for bortezomib refractory patients or in terms of bortezomib-based combinations.

Thalidomide, is the first IMiD used for the treatment of myeloma patients. Due to the fact that thalidomide is not excreted by kidneys no dose adjustment is needed in patients with RI. When combined with high-dose dexamethasone 55-75% improved their renal function in the newly diagnosed setting [1] and approximately 60% in the relapsed refractory setting [2]. Furthermore, the combination with bortezomib is highly effective and common for NDMM with RI especially in Europe. For patients undergoing dialysis close monitoring is essential due to unexpected hyperkalemia.

Lenalidomide although similar to thalidomide is renaly excreted and therefore the relevant dose adjustments are based on the extent of RI. When adjusted lenalidomide is both effective and safe and some patients will even improve their renal function. In the phase 3 MM-009 and MM-010 trials, 353 patients were randomized to receive the combination lenalidomide and dexamethasone (RD). Among them 98 were identified with RI (eGFR<60ml/min) and this subgroup had similar overall response rate (ORR) and progression free survival (PFS) rate with the non-RI group, while most importantly renal improvement was achieved in 72%. These patients mainly experienced lenalidomide-induced thrombocytopenia and in 15 of them dose reductions were required. The study design did not include dose adjustments for lenalidomide according to CrCl and all patients with CrCl<50 ml/min received full dose [3]. A prospective phase II study evaluated the efficacy of dose adjusted RD in 35 patients with acute FLC-induced renal failure. Myeloma response was observed in 69% and renal response was observed in 45.6%. 5 out of 13 (38%) patients became dialysis independent [4]. In another study, 1 patient out of 15 dialysis-dependent RRMM patients, became independent after RD. The use of lenalidomide remains challenging in severe RI, however can be used especially whenever bortezomib-based therapy is not feasible.

Pomalidomide is a new generation IMiD approved by the FDA for the treatment of RRMM patients who have received at least two previous lines of therapy. Only 2% of pomalidomide is renaly excreted and until recently available data on dosing adjustment, safety, and toxicity profile was limited because patients with RI were excluded from clinical trials. However, dose adjustments are not necessary in patients with mild or moderate RI (CrCl >=45ml/min).113 patients received pomalidomide plus low-dose dexamethasone in a retrospective analysis of a phase I/II trial. The toxicity profile was similar across groups with different RI degree [5]. In the MM-002 sub-analysis the patients were stratified according to CrCl and both efficacy and safety were similar between the different groups. In the phase III MM-03 trial patients with CrCl <45ml/min were excluded, however PFS, overall survival (OS) and toxicity were similar among patients with normal renal function and CrCl<60m/min [6]. Similarly, in the phase IIIb STRATUS trial the toxicity profile of pomalidomide was same in all patients groups [7]. Prospective phase II MM-013 trial was designed in order to assess efficacy and safety of pomalidomide and low-dose dexamethasone in RRMM patients with moderate, severe and end-stage renal disease (eGFR < 45 mL/min/1.73 m2 )[8]. The enrolled patients were enrolled in 3 renal cohorts: cohort A—moderate renal impairment (eGFR 30 to < 45 mL/min/1.73 m2), cohort B—severe renal impairment (eGFR < 30 mL/min/1.73 m2), and cohort C—severe renal impairment requiring hemodialysis. The results of the study were recently published and demonstrated that pomalidomide at the 4 mg/day dose level plus low dose dexamethasone was effective and safe in all 3 cohorts, while no new safety signals were observed.

In conclusion, the introduction of novel agents has improved RI reversal leading to favorable outcomes in most patients as long as the appropriate treatment regimen ispromptly administered. Bortezomib remains the cornerstone in RI management, however in high risk patients triplet combinations are highly recommended. IMiDs have been associated with some degree of renal recovery with manageable toxicity profile and should be considered especially for bortezomib-refractory patients. Treatment choices should be individualized and based on previous lines of therapy, and specific patients and disease characteristics.
